# High frequency ultrasound in breast surgery: A narrative review of current evidence and future directions

**DOI:** 10.1016/j.jpra.2026.08.020

**Published:** 2026-08-20

**Authors:** David Kwan Ryung Lee, Dave Osinachukwu Duru, Kai Yuen Wong

**Affiliations:** a School of Clinical Medicine, University of Cambridge, Hills Rd, Cambridge, United Kingdom; b Department of Plastic and Reconstructive Surgery, Cambridge University Hospitals NHS Foundation Trust, Cambridge, United Kingdom

**Keywords:** High-frequency ultrasound, Breast surgery, Plastic surgery, Breast implants, Microsurgical planning

## Abstract

**Background:**

High-frequency ultrasound (HFUS) is a versatile but underutilised imaging modality in breast surgery. Its high resolution offers a unique imaging perspective when compared to other modalities such as magnetic resonance imaging (MRI) and conventional, lower frequency ultrasound (<10 MHz). This narrative review synthesises current literature on the applications of HFUS in breast surgery including future directions.

**Methods:**

A comprehensive search of PubMed, Embase, Web of Science, CiNAHL, Cochrane CENTRAL and Scopus was conducted for studies published between January 2020 and September 2025. Eligible articles included clinical studies, reviews, and technical evaluations using probes >10 MHz in breast surgery applications.

**Results:**

HFUS demonstrates strong clinical utility in implant monitoring, lymphatic supermicrosurgery and breast reconstruction. Compared to MRI, emerging evidence indicates that HFUS can produce concordant results in 87–100% of cases, whilst displaying additional advantages such as greater accessibility and real-time imaging. The high definition offered by HFUS facilitates flap reconstruction planning, and its ability to visualise lymphatic channels and dermal thickness is also a useful tool for the planning of lymphaticovenous anastomoses for lymphoedema.

**Conclusion:**

HFUS imaging appears to show great promise for breast surgery, although future research on comparative studies to develop imaging protocols is needed. More widespread adoption will also require integration into surgical training curricula.

## Introduction

Ultrasound is a fundamental imaging modality with wide-ranging applications across medical and surgical disciplines. By adjusting the frequency of ultrasound waves, clinicians can optimise the balance between tissue penetration and spatial resolution. Ultrasound typically operates within a frequency range of 1–15 MHz.^1^ Although no universal consensus exists, high frequency-ultrasound (HFUS) is typically defined as the use of probes at the upper end of this range – most commonly 10 MHz and above.^2^, ^3^, ^4^, ^5^, ^6^, ^7^ These higher frequencies provide superior spatial resolution but are limited by shallow tissue penetration. For example, 10 MHz probes can theoretically distinguish between points up to 0.23 mm apart.^8^ Ultra-high-frequency ultrasound (UHFUS) operates at even higher frequencies, usually between 48 MHz and 70 MHz, and can achieve a finer resolution of 0.03 mm.^9^^,^^10^ Note however, that the use of these ultra-high frequency probes is restricted by shallow imaging depths of 3–4 mm, in comparison to the 35 mm of tissue penetration achieved when using 10 MHz.^11^

The high spatial resolution of HFUS is used for applications such as monitoring dermal filler integration,^12^ guiding facial injections to avoid vascular compromise,^13^ flap planning,^14^^,^^15^ and lymphatic reconstructions.^3^ This narrative review summarises aspects of breast surgery that may be enhanced by HFUS, as well as potential future directions.

## Materials and methods

A comprehensive literature search was conducted in PubMed, Embase, Web of Science, CiNAHL, Cochrane CENTRAL and Scopus for articles published between January 2020 and September 2025. This included the use of keywords such as “plastic surge*”, “breast surg*” and “high-frequency ultrasound” – see Supplementary Figure A for the full search strategy. Papers were screened by two independent reviewers (D.K.R.L.) and (D.O.D.) using Rayyan AI Software. Eligible studies included original experimental or observational research as well systematic, literature and narrative reviews. Relevant references were also screened from included articles. Only studies with primary patient data (n ≥ 5), explicitly reporting the use of HFUS probes that could reach frequencies >10 MHz for breast-related applications in plastic surgery, were included. Exclusion criteria were conference abstracts, studies employing conventional ultrasound frequencies ≤10 MHz, non-human research, case reports and publications not available in English.

## Search results

**Fig. 1:**
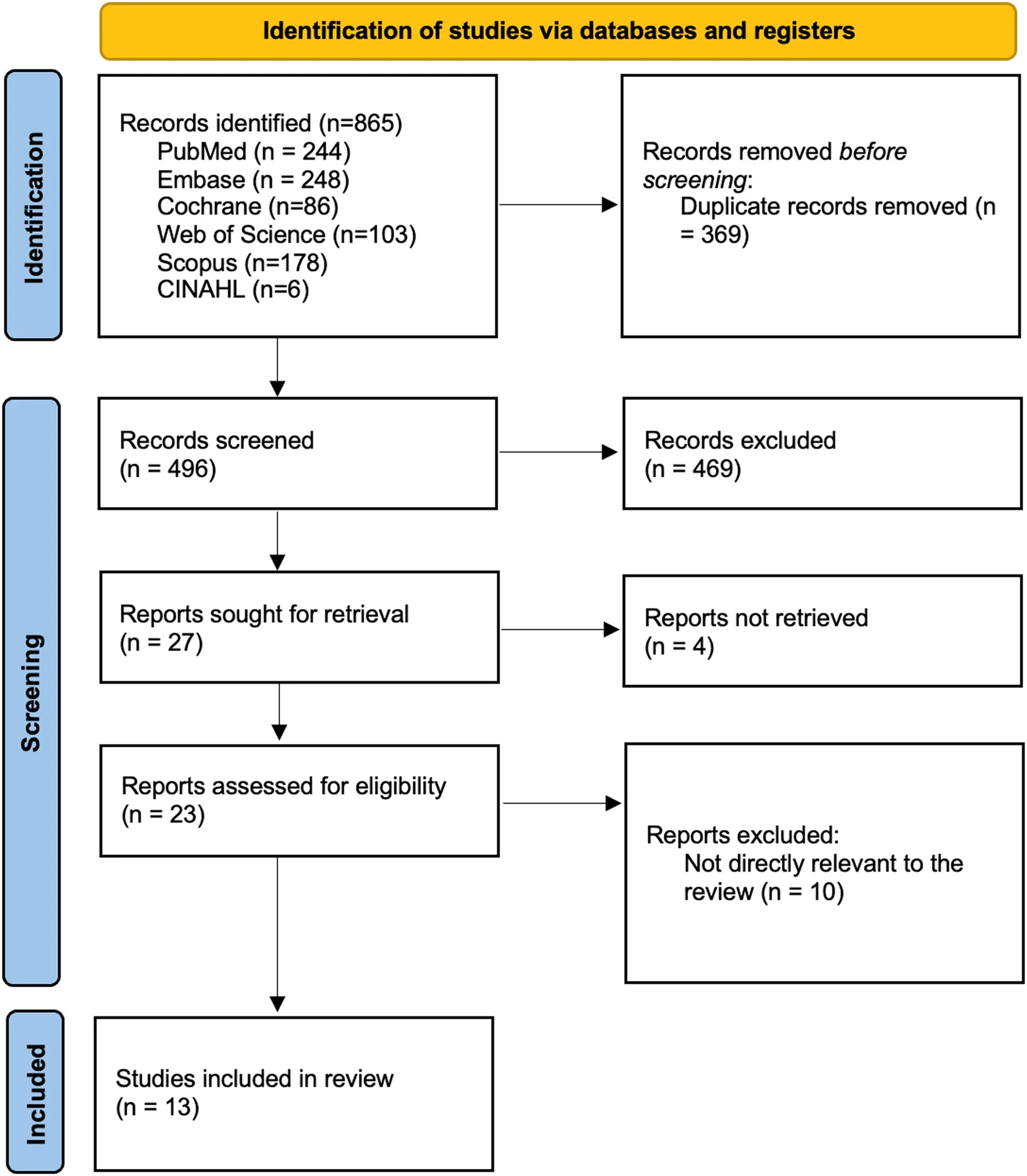
PRISMA flow diagram indicating the paper search results.

A total of 865 studies were initially identified, with a total of 13 studies being included after duplication removal and screening. Fig. 1 demonstrates a PRISMA flow chart of this process and Table 1 demonstrates their specific characteristics. Studies reporting the use of ultrasound probes that extended over a range of frequencies that were both above and below 10 MHz were included. Consequently, a limitation to consider involves the possibility that surgeons may not have always used an ultrasound frequency that was greater than 10 MHz in the reported studies.

**Table 1:** Characteristics of studies included.

Author	Year	Study Design	HFUS Application	HFUS Frequency (MHz)
Diesch et al^22^	2022	Experimental in vitro Comparative Study	Implant Monitoring	6–15, 9–16, 12.5–33
Bang et al^24^	2022	Retrospective Diagnostic Accuracy Study	Implant Monitoring	7–18
Chai et al^27^	2025	Retrospective Cross-Sectional Descriptive Study	Implant Monitoring	15
Kim et al^32^	2023	Retrospective Cross-Sectional Descriptive Study	Implant Monitoring	7–18
Nam et al^33^	2022	Retrospective Descriptive Study	Implant Monitoring	7–18
Visconti et al^36^	2024	Retrospective Comparative Cohort Study	Breast Reconstruction	12,18
Hong et al^38^	2025	Retrospective Case Series	Breast Reconstruction	10–18
Angrigiani et al^39^	2021	Retrospective Case Series	Breast Reconstruction	5–12
Kuo et al^40^	2025	Prospective Case Series	Breast Reconstruction	48 (UHFUS)
Chew et al^42^	2024	Technical Report+Case Series	Breast Reconstruction	9–15
Hashem and Yamamoto^49^	2025	Retrospective Observational Cohort Study	Lymphoedema Management	18
Kilbreath et al^54^	2022	Secondary Analysis of Randomized Control Trial	Lymphoedema Management	6–18
Malagon et al^55^	2025	Cross-sectional Case Control Study	Lymphoedema Management	70 (UHFUS)

## Applications of HFUS in breast surgery

### Breast implants

The current gold standard imaging for implant evaluation is magnetic resonance imaging (MRI).^16^ It offers a high diagnostic sensitivity and specificity of 83–100%^17^^,^^18^ and 90–98%^18^^,^^19^ respectively and offers recognised signs such as the ‘linguini sign’ that indicates intracapsular rupture.^20^ However, this imaging modality has several limitations including the fact that it can be costly, time consuming, and is contraindicated in certain patients with MR-unsafe devices.

On the other hand, ultrasound is more widely available, cheaper and scans can be completed quicker when monitoring implants.^16^ However, evidence suggests that when using frequencies between 1 and 10 MHz, it is less accurate than MRI, displaying sensitivities and specificities of 67.0–85.2% and 73.0–96.1% respectively.^17^^,^^19^^,^^21^ HFUS has all the accessibility benefits of conventional ultrasound, but may also demonstrate higher sensitivity and specificity rates than its lower-frequency counterpart due to its higher resolution.

Diesch et al. (2022)^22^ analysed the resolution of implants produced by probes of 6–15 MHz, 9–16 MHz, and 12.5–33 MHz. The resolution produced by 12.5–33 MHz probes was significantly higher than the rest and was therefore recommended for routine implant monitoring – although a significant limitation of this study is that it was effectuated in vitro. Nonetheless, this higher frequency range is further supported by additional in vivo studies such as that of Lake et al. (2013),^23^ who recommends using a minimum of 12 MHz when monitoring implants. They believe that these higher resolutions are required to observe key implant rupture signs such as shell membrane/capsular breaches, debris signs and echogenic folds.

Of note, HFUS appears to show a higher reported sensitivity and specificity up to 98.3% and 99% respectively for detecting implant ruptures in patients – see Table 2. In addition, when comparing HFUS to MRI, Hold et al. (2012)^18^ reported concordant results 87% of the time when assessing 60 implants from 34 patients, whereas Bengtson and Eaves (2012)^16^ reported 100% concordance assessing 29 implants from 15 patients. Bang et al. (2022)^24^ reported 100% concordance between HFUS and intra-operative findings when diagnosing implant rupture.

**Table 2:** Sensitivity and specificity for each imaging modality for implant rupture.

Imaging Modality	Authors	Year	Frequency (MHz)	Sensitivity (%)	Specificity (%)
MRI	Ikeda et al^17^	1999	N/A	100.0	63.0
MRI	Rietjens et al^19^	2014	N/A	83.0	98.0
Conventional Ultrasound	Liston et al^59^	1994	5.0	70.0	96.0
Conventional Ultrasound	Ikeda et al^17^	1999	7.5	67.0	92.0
Conventional Ultrasound	Rietjens et al^19^	2014	8.0–10.0	69.0	73.0
Conventional Ultrasound	Ferenz et al^21^	2023	1.0–10	85.2	96.1
HFUS	Hold et al^18^	2012	11.0	50.0	90.0
HFUS	Rukanskiene et al^25^	2021	12.0	98.3	89.2
HFUS	Bengtson^26^	2021	12.0–18.0	97.0	99.0

However, there are key considerations to be made when evaluating the results put forward by Table 2. Results of all imaging modalities will be significantly different depending on their users. Furthermore, technological differences, regarding both imaging devices and implants, may contribute to study heterogeneity, given that studies from 1994 to 2023 were shown. For example, Bengston and Eaves (2012)^16^ also suggest that the accuracy of HFUS may also depend on implant type, with newer implants providing clearer imaging. Of note, Hold et al. (2012)^18^ used 11 MHz probes, yet reported a distinctively lower sensitivity of 50%, compared to Rukanskiene et al. (2021)^25^ and Bengtson et al. (2021).^26^ Indeed, a limitation highlighted by Hold et al. was that all ruptured implants were older, with various unknown manufacturers being reported. Overall, although promising, further definitive analyses between HFUS, conventional ultrasound and MRI outcomes are necessary.

HFUS has also been used to visualise pathologies associated with breast fillers. Chai et al. (2025)^27^ used 15 MHz probes to successfully identify complications such as fat necrosis and cyst formation. Although sensitivity and specificity evaluations were not carried out, the authors did note that the high resolution provided by HFUS probes allowed clear visualisation of blocked blood vessels and therefore signs of expander implantation/filler infection in the breast. Higher quality evidence evaluating HFUS’s specificity/sensitivity for detecting breast filler pathology is needed.

Macrotextured implants have been shown to have a causal relationship with both capsular contraction and BIA-ALCL.^28^, ^29^, ^30^, ^31^ Kim et al. (2023)^32^ found that the high resolution offered by HFUS gives breast surgeons the ability to distinguish between smooth and macrotextured implants, so that recommended removal can take place. Furthermore, they were also able to successfully categorise implants amongst the eight manufacturers included in the study, given that each manufacturer demonstrated a unique ultrasound image. Similarly, Nam et al. (2022)^33^ used 7–18 MHz probes and found 100% concordance for 136 implants between HFUS and intraoperative findings when it came to surface texture, shape and manufacturer. Ultimately, implant types or manufacturers that are strongly associated with pathologies can be easily identified using the high-definition images of HFUS, making it a valuable tool for breast surgery guidance.

Limitations of HFUS that prevent it from reaching 100% sensitivity may include technical artefacts such as reverberations and radial folds, which are common and can be misinterpreted as signs of intracapsular rupture.^22^ Acoustic shadowing, particularly in the presence of dense fibrous capsules, may obscure deeper structures, thereby limiting diagnostic confidence.^34^ Finally, posterior portions of breast implants are more difficult to visualise than anterior aspects, where image resolution and sensitivity are diminished.^22^

Despite these limitations, some data suggests that incorporating ultrasound into imaging strategies may be more cost-effective – a cost-analysis by Chung et al. (2013)^35^ recommends an economically optimal approach of using ultrasound and MRI for asymptomatic women, and ultrasound alone for symptomatic women. Whether this remains the case for more expensive, higher-resolution HFUS devices has yet to be evaluated.

### Breast reconstruction

HFUS is also useful for free flap breast reconstruction planning by mapping out the course of the perforators^36^ and hence minimising surgical morbidity.^37^ HFUS has been noted to be highly effective when mapping perforators of the thoracodorsal artery,^38^ anterior intercostal artery,^39^ superficial inferior epigastric vessels^40^^,^^41^ and deep inferior epigastric vessels.^42^^,^^43^

HFUS can visualise vessels <0.8 mm, so it also allows planning of true perforator flaps. A perforator is defined to be a vessel that perforates an envelope of a targeted tissue. It allows for selective harvesting of tissue, thereby minimising donor site morbidity and allowing consideration of any donor site for harvest.^44^ Visconti et al. (2024)^36^ used 18 MHz probes to design true thoracodorsal artery perforator, lateral intercostal artery perforator and lateral thoracic artery perforator flaps, which significantly reduced their operation times.

### Breast-Related lymphatic surgery

Indocyanine green (ICG)-based lymphography is the current standard for surgical planning of lymphaticovenous anastomosis (LVA), which is used for the treatment and prevention of lymphoedema.^45^^,^^46^ The high resolution of HFUS allows direct visualisation of lymphatic channels so can also be used for lymphatic reconstruction planning.^47^^,^^48^ For example, Hashem and Yamamoto used 18 MHz probes (2025)^49^ for lymphatic vessel evaluation in 50 out of 54 (92.6%) LVA procedures. Furthermore, statistically significant improvements were observed in limb volume, heaviness, tension, cellulitis frequency and mobility. Additional studies have also shown that with adequate practice, rates of lymphatic vessel identification using HFUS may be higher and up to 100%^50^, ^51^, ^52^, ^53^ in both upper and lower limb LVAs.

The high resolution of HFUS can also be used to evaluate the extent of post-surgical oedema in breast cancer patients. Kilbreath et al. (2022)^54^ suggested that a change in dermal thickness of magnitude 0.34–0.41 mm, detectable using only the higher frequencies of 6–18 MHz, may significantly indicate breast oedema. Similarly, Malagon et al. (2025)^55^ found that an increase of 0.28 mm of breast dermal thickness, measured using 70 MHz UHFUS, showed 100% concordance when compared to diagnosing lymphoedema using ICG lymphography. Although further work investigating the correlation between these monitored changes and patient reported outcomes is needed, HFUS could possibly be used to detect the changes in skin thickness for monitoring oedema.

### Clinical incorporation of HFUS

HFUS, like many imaging modalities, has already been established to be highly operator-dependent.^56^ Fortunately, evidence suggests that even a one-day training session can substantially improve a clinician’s competency in ultrasound,^57^ with further skills often acquired through supervised clinical practice.^58^ Ferenz et al. (2023)^21^ found that there was a significant improvement in sensitivity, per 10 cases of HFUS examination. Moreover, they found that after 60 sessions, sensitivity scores that were higher than what is typically reported in the literature were reached.

In addition, Hong et al. (2025)^38^ report similar findings, showing that the first seven patients to receive HFUS-assisted breast flap reconstruction had significantly longer operation times by 47 min on average, compared to the last seven patients. The authors attributed the time saved due to a combination of both the increased familiarity of HFUS and the surgical dissection itself. Therefore, incorporation of HFUS into the clinical setting would appear to benefit from likewise incorporation into surgical training curricula. Further evidence supporting specific teaching guidelines is required.

## Conclusion

HFUS is a useful yet underutilised tool in breast plastic surgery. Its relative affordability, portability, and capacity for real-time, high-resolution imaging make it particularly well suited to a range of clinical applications. HFUS shows efficacy for implant evaluation, frequently correlating with MRI and intraoperative findings. It also facilitates planning of microsurgical and lymphatic reconstructions. The integration of HFUS into formal plastic surgery training curricula is both timely and necessary. Further studies are required to evaluate its cost-effectiveness and effect on patient reported outcomes.

## Funding

None.

## Ethical approval

Not required.

## Presentations

This paper has been accepted for poster presentation at Association of Surgeons in Training (ASiT) 2026.

## Financial disclosure statement

The authors have nothing to disclose.

## Conflicts of interest

None declared.

## Appendix Supplementary materials

Image, application 1
