# Supplementary material for: High frequency ultrasound in breast surgery: A narrative review of current evidence and future directions
Source: JPRAS Open. 2026 Aug 20;52:154–60. doi: 10.1016/j.jpra.2026.08.020 (PMC13563231; doi:10.1016/j.jpra.2026.08.020)
Supplement: Supplementary file 1 [file mmc1.docx]

**Supplementary Figure A:**

Pubmed:

"Ultrasonography"[Mesh] OR "Ultrasonics"[Mesh] OR “ultraso*”[tiab ] OR “High-frequency ultraso*”[tiab] OR “Ultra-high-frequency ultraso*”[tiab] OR "High-resolution ultrasound"[tiab] OR "HFUS"[tiab] OR "HRUS"[tiab] OR "UHFUS"[tiab]

AND

"Plastic Surgery Procedures"[Mesh] OR "Plastic Surge*"[tiab] OR "Reconstructive Surge*"[tiab]

AND

"Breast"[Mesh] OR "Breast Implants"[Mesh] OR "Breast Implantation"[Mesh] OR "Mastectomy"[Mesh] OR "Mammaplasty"[Mesh] OR "Breast*"[tiab] OR "Breast Implant*"[tiab] OR "Mastectom*"[tiab] OR "Mammaplast*"[tiab]

Embase:

| **#** | **Query** |
| --- | --- |
| 1 | echography/ |
| 2 | ultrasound/ |
| 3 | ultraso*.mp. |
| 4 | high frequency ultrasound/ |
| 5 | High-frequency ultraso*.mp. |
| 6 | Ultra-high-frequency ultraso*.mp. |
| 7 | High-resolution ultraso*.mp. |
| 8 | HFUS.mp. |
| 9 | HRUS.mp. |
| 10 | UHFUS.mp. |
| 11 | 1 or 2 or 3 or 4 or 5 or 6 or 7 or 8 or 9 or 10 |
| 12 | plastic surgery/ |
| 13 | plastic surg*.mp. |
| 14 | reconstructive surgery/ |
| 15 | reconstructive surge*.mp. |
| 16 | 12 or 13 or 14 or 15 |
| 17 | breast/ or breast reduction/ or breast reconstruction/ or breast surgery/ |
| 18 | breast endoprosthesis/ |
| 19 | mastectomy/ |
| 20 | breast augmentation/ |
| 21 | breast*.mp. |
| 22 | breast implant*.mp. |
| 23 | mastectom*.mp. |
| 24 | mammaplast*.mp. |
| 25 | 17 or 18 or 19 or 20 or 21 or 22 or 23 or 24 |
| 26 | 11 and 16 and 25 |

Cochrane:

ID	Search	Hits

#1	MeSH descriptor: [Ultrasonography] explode all trees

#2	ultraso*

#3	high-frequency ultraso*

#4	ultra-high-frequency ultraso*

#5	high-resolution ultrasound

#6	HFUS

#7	HRUS

#8	UHFUS

#9	#1 OR #2 OR #3 OR #4 OR #5 OR #6 OR #7 OR #8

#10	MeSH descriptor: [Surgery, Plastic] explode all trees

#11	MeSH descriptor: [Plastic Surgery Procedures] explode all trees

#12	plastic surge*

#13	reconstructive surge*

#14	#10 OR #11 OR #12 OR #13

#15	MeSH descriptor: [Breast] explode all trees

#16	MeSH descriptor: [Breast Implants] explode all trees

#17	MeSH descriptor: [Breast Implantation] explode all trees

#18	MeSH descriptor: [Mastectomy] explode all trees

#19	MeSH descriptor: [Mammaplasty] explode all trees

#20	breast*

#21	breast implant*

#22	mastectom*

#23	mammaplast*

#24	#15 OR #16 OR #17 OR #18 OR #19 OR #20 OR #21 OR #22 OR #23

#25	#9 AND #14 AND #24

Web of Science:

TS=(ultraso* OR HFUS OR HRUS OR UHFUS)

AND  TS=("plastic surge* OR reconstructive surge*)

AND

TS=(breast OR "breast implant* OR mastectom* OR mammaplast*)

Scopus:

( TITLE-ABS-KEY ( ultraso* OR HFUS OR HRUS OR UHFUS ) AND TITLE-ABS-KEY ( "plastic surge*" OR "reconstructive surge*") AND TITLE-ABS-KEY ( breast OR "breast implant*" OR mastectom* OR mammaplast* ) ) AND PUBYEAR > 2019 AND PUBYEAR < 2026

CINAHL:

S1 XB(ultraso* OR HFUS OR HRUS OR UHFUS)

S2 XB(“plastic surge*” OR “reconstructive surge*”)

S3 XB(breast OR “breast implant*” OR mastect* OR mammaplasty*)

S4 S1 AND S2 AND S3
